# PLGA-PEG Nanoparticles Loaded with Cdc42 Inhibitor for Colorectal Cancer Targeted Therapy

**DOI:** 10.3390/pharmaceutics16101301

**Published:** 2024-10-06

**Authors:** Sanazar Kadyr, Altyn Zhuraliyeva, Aislu Yermekova, Aigerim Makhambetova, Daulet B. Kaldybekov, Ellina A. Mun, Denis Bulanin, Sholpan N. Askarova, Bauyrzhan A. Umbayev

**Affiliations:** 1 School of Medicine, Nazarbayev University, 010000 Astana, Kazakhstan; sanazar.kadyr@nu.edu.kz (S.K.); dbulanin@nu.edu.kz (D.B.); 2 Laboratory of Bioengineering and Regenerative Medicine, National Laboratory Astana, Nazarbayev University, 010000 Astana, Kazakhstan; altyn.zhuraliyeva@nu.edu.kz (A.Z.); aislu.yermekova@nu.edu.kz (A.Y.); aigerim.makhambetova@nu.edu.kz (A.M.); shaskarova@nu.edu.kz (S.N.A.); 3 Department of Chemistry and Chemical Technology, Al-Farabi Kazakh National University, 050040 Almaty, Kazakhstan; dauletchem@gmail.com; 4 School of Sciences and Humanities, Nazarbayev University, 010000 Astana, Kazakhstan; ellina.mun@nu.edu.kz

**Keywords:** Cdc42, CASIN, colorectal cancer, PLGA-PEG-COOH, nanoparticles

## Abstract

**Background/Objectives:** An inhibitor of small Rho GTPase Cdc42, CASIN, has been shown to reduce cancer cell proliferation, migration, and invasion, yet it has several limitations, including rapid drug elimination and low bioavailability, which prevents its systemic administration. In this study, we designed and characterized a nanoparticle-based delivery system for CASIN encapsulated within poly(lactide-co-glycolide)-block-poly(ethylene glycol)-carboxylic acid endcap nanoparticles (PLGA-PEG-COOH NPs) for targeted inhibition of Cdc42 activity in colon cancer. **Methods:** We applied DLS, TEM, and UV–vis spectroscopy methods to characterize the size, polydispersity index, zeta potential, encapsulation efficiency, loading capacity, and in vitro drug release of the synthesized nanoparticles. The CCK-8 cell viability test was used to study colorectal cancer cell growth in vitro. **Results:** We showed that CASIN-PLGA-PEG-COOH NPs were smooth, spherical, and had a particle size of 86 ± 1 nm, with an encapsulation efficiency of 66 ± 5% and a drug-loading capacity of 5 ± 1%. CASIN was gradually released from NPs, reaching its peak after 24 h, and could effectively inhibit the proliferation of HT-29 (IC50 = 19.55 µM), SW620 (IC50 = 9.33 µM), and HCT116 (IC50 = 10.45 µM) cells in concentrations ranging between 0.025–0.375 mg/mL. CASIN-PLGA-PEG-COOH NPs demonstrated low hemolytic activity with a hemolytic ratio of less than 1% for all tested concentrations. **Conclusion:** CASIN-PLGA-PEG-COOH NPs have high encapsulation efficiency, sustained drug release, good hemocompatibility, and antitumor activity in vitro. Our results suggest that PLGA-PEG-COOH nanoparticles loaded with CASIN show potential as a targeted treatment for colorectal cancer and could be recommended for further in vivo evaluation.

## 1. Introduction

Colorectal cancer (CRC) is a prevalent and significant public health issue, with an estimated 1.93 million new cases diagnosed worldwide in 2020, which are projected to reach 3.2 million by 2040 [1,2]. At early stages, surgical treatment alone or combined with chemotherapy and radiation therapy is the primary type of CRC therapy [3,4]. However, systemic chemotherapy’s effectiveness can vary, leading to relapses due to resistance or tumor progression [4]. The study of new targeted molecules that specifically interact with specific cell receptors or proteins and inhibit cancer cells’ growth, progression, and invasiveness is crucial for developing new effective anticancer therapies [5,6].

Currently, six targeted drugs are used for the therapy of CRC: anti-VEGF drugs such as bevacizumab, ramucirumab, and ziv-aflibercept; anti-EGFR drugs such as cetuximab and panitumumab; and the drug multikinase inhibitor regorafenib [7]. Despite the significant progress made in the development of targeted drugs, an important factor limiting the effectiveness of targeted therapy is the presence of subpopulations of cells with different genotypes and phenotypes in a tumor, the so-called heterogeneity of tumors [5]. Tumor heterogeneity leads to distinct tumor behavior in patients with the same type of tumor during therapy [8]. Therefore, many approved inhibitors cannot be used in most patients, and new targets must be identified for more individualized treatment [8].

The small Rho GTPase, cell division control protein 42 homolog (Cdc42), has recently been proposed as a promising agent for targeted cancer therapy [9,10]. Cdc42 is a highly conserved protein crucial in intercellular adhesion, cytoskeletal structure formation, and cell cycle regulation [11]. The overexpression of Cdc42 has been found in various cancer types, such as breast cancer, testicular cancer, head and neck squamous cell carcinoma, melanoma, and CRC [12]. In human CRC, a high expression of Cdc42 in tumor cells was noted in 60% of cases [10].

The oncogenic role of Cdc42 in CRC is mediated by the transcriptional dysregulation of key genes and pathways associated with cell proliferation and the neoplastic transformation of KRAS [13]. Valdés-Mora et al. showed that an overexpression of Cdc42 and suppression of the tumor suppressor gene CACNA2D2 is associated with the most aggressive subgroup of CRC [13]. Several studies have examined the effects of Cdc42 inhibition on CRC cells using small molecules [9,14]. It was shown that the Cdc42 inhibitor AZA197 suppressed the proliferation, migration, and invasion of SW620 and HT-29 colon cancer cells and increased apoptosis [14]. Also, an in vivo study showed that AZA197 reduced tumor xenograft growth based on the SW620 line by inhibiting cell proliferation and inducing apoptosis [14].

The specific inhibitor of Cdc42, CASIN, decreased the oncogenicity of four Cdc42-overexpressing colorectal cancer cell lines (LIM1863, Caco2, LIM1899, and LIM2551) in vitro [9]. In addition, LIM1863 treated with 10 µM CASIN for 16 h showed reduced carcinogenicity in a xenograft model [9]. CASIN is a commercially available specific small molecule inhibitor of Cdc42 with submicromolar affinity [15], low in vivo toxicity [16], and high anticancer activity [12], which makes it an attractive potent anticancer drug. However, the hydrophobicity, low bioavailability, and rapid excretion rate of the molecule restrict the therapeutic use of CASIN [15]. In addition, the activity of this enzyme is also increased in some physiological conditions, limiting its systemic use [11]. In this regard, developing a nanoparticle-based delivery system for CASIN might circumvent these problems by boosting medication accumulation in tumor tissue and reducing systemic side effects.

Since CASIN is a hydrophobic molecule [17], PLGA-PEG nanoparticles could significantly improve its pharmacokinetic properties and bioavailability. PLGA microspheres are biodegradable polymers known for their excellent biocompatibility and non-toxicity. They can form membranes and capsules, making them widely utilized in drug delivery and release systems and tissue engineering scaffolds [18,19]. Polyethylene glycol (PEG) is introduced to the nanoparticle structure to form a PEG corona on the surface of polymer nanoparticles and enhance the permeation of the CASIN delivery system through the mucosal layer surrounding tumor tissues in the colon [20]. In addition, PLGA-PEG nanoparticles are FDA-approved [21].

In this regard, we have designed a novel Cdc42 inhibitor nanoformulation in the present work by encapsulating the Cdc42 inhibitor CASIN within PLGA-PEG-COOH nanoparticles (NPs) using the one-step nanoprecipitation (single-emulsion) method. The particle size, zeta potential, and in vitro release properties of CASIN-loaded PLGA-PEG-COOH NPs were studied. The cytotoxic effects against human colorectal carcinoma cells and the hemocompatibility of CASIN-loaded PLGA-PEG-COOH NPs were also investigated. To our knowledge, no previously published study has investigated the proposed drug delivery system for CRC therapy based on a Cdc42 inhibitor encapsulated in PLGA-PEG nanoparticles.

## 2. Results and Discussion

### 2.1. Preparation and Characterization of Nanoparticles

PLGA-PEG-COOH nanoparticles, with and without CASIN, were developed using a slightly modified single-emulsion technique based on a previously published report [22]. This is a common and very efficient technique for formulating NPs using block copolymers of two or more polymer chains with different hydrophobicity. These copolymers spontaneously self-assemble into a core-shell structure in an aqueous environment. Polymeric micelles or nanocarriers formed this way can solubilize and deliver hydrophobic substances, thereby protecting them from premature degradation and enhancing their bioavailability [20,23].

**Figure 1:**
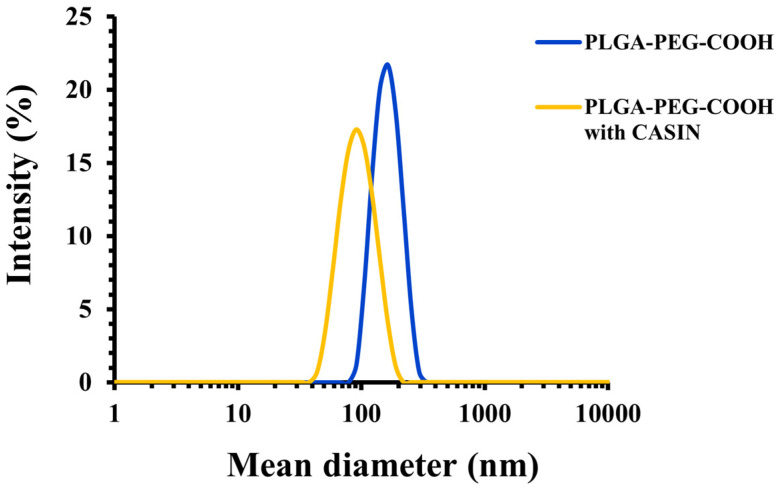
Size distribution of PLGA-PEG-COOH nanoparticles with and without CASIN as determined by DLS.

The physicochemical characteristics of PLGA-PEG-COOH nanoparticles, including their encapsulation efficiency and loading capacity, are summarized in Table 1. It can be seen that the loading of PLGA-PEG-COOH NPs with CASIN led to a significant near two-fold decrease in nanoparticle diameter compared to CASIN-free NPs. Moreover, both unloaded and CASIN-loaded NPs displayed high negative zeta-potential (≤−30 mV), ensuring excellent colloidal stability and a low polydispersity of <<0.20, indicating the presence of a homogeneous population with a narrow size distribution (Figure 1). The high CASIN encapsulation efficiency of 66 ± 5% indicates successful loading (Table 1). The drug-loading capacity is 5 ± 1% *w*/*w*, which means that 5% of the mass of the nanoparticles consists of CASIN, which is a favorable level for therapeutic use [24]. Additional data from other NP batches are summarized in Table S1 in the Supplementary Information.

**Figure 2:**
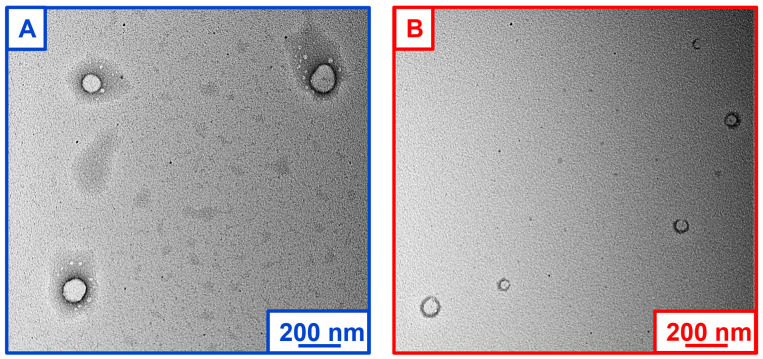
TEM microphotographs of PLGA-PEG-COOH nanoparticles: empty (**A**) and CASIN-loaded (**B**).

The size and morphology of the developed CASIN-loaded PLGA-PEG nanoparticles were further studied using transmission electron microscopy (TEM) and microphotographs (Figure 2). The nanoparticles were stained with a 2% uranyl acetate alternative to achieve reasonable contrast for imaging. The analysis of TEM images of empty and CASIN-loaded PLGA-PEG-COOH nanoparticles revealed the formation of well-dispersed spherically shaped homogeneous structures with a smooth surfaces. The average size estimated by microscopy agrees with the data acquired by DLS measurements (Table 1). Additional images to further illustrate TEM images are depicted in Figure S2 included in the Supplementary Materials.

**Figure 3:**
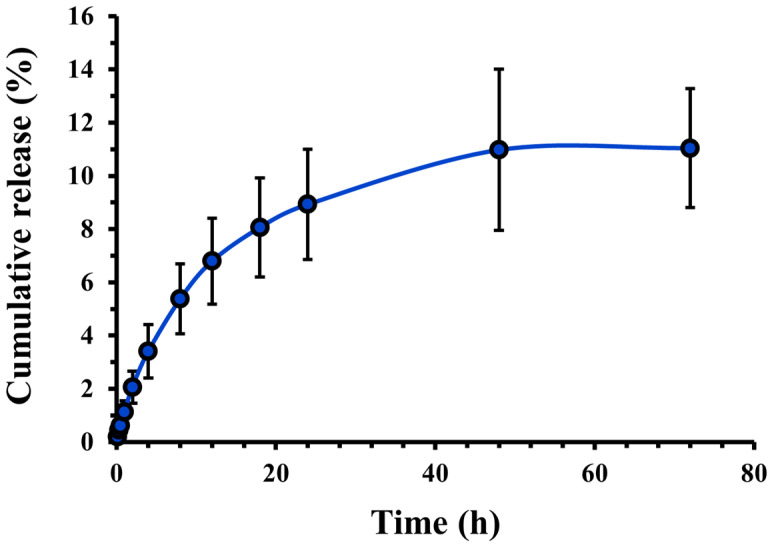
Cumulative release profile of CASIN from PLGA-PEG-COOH nanoparticles. Data expressed as mean ± standard deviation (*n* = 3).

The in vitro release study results are the cumulative percentage of drugs released over 72 h (Figure 3). The release of CASIN from PLGA-PEG-COOH nanoparticles occurred gradually and reached a maximum after 24 h. The release continued over the next 48 h, although its rate decreased sharply, indicating a consistent and controlled release. The dynamics of this release are essential to ensure consistent therapeutic effects. The drug’s rapid initial distribution quickly approaches therapeutic concentrations, which may be a key to targeting cancer cells. The gradual release of the drug over subsequent hours ensures that the required drug level is maintained, which reduces the frequency of repeated dosing and limits the potential adverse effects associated with high peak concentrations. This release profile fits well with the Higuchi kinetics model (R^2^ = 0.9427) and the Korsmeyer Peppas model (R^2^ = 0.9658) (Figure S3), which describes the drug dissolution in pharmaceutical dosage forms containing water-insoluble drugs in hydrophobic–hydrophilic matrices or polymeric nanoparticle suspension/micelles. This may be a consequence of additional polymer inclusion that is used to stabilize/coat the NPs (Poloxamer 407), resulting in a gradual release [25].

### 2.2. Cytotoxicity Effect of NPs

As mentioned above, Cdc42 inhibition could suppress the tumorigenicity of human colorectal cancer cells with higher levels of Cdc42 expression [9]. On the other hand, measuring Cdc42 activity rather than expression may offer new insights into the role of Cdc42 dysregulation in cancer development [26]. Therefore, in the present study, we evaluated the cytotoxicity of CASIN-loaded PLGA-PEG-COOH nanoparticles in SW620, HCT116, and HT-29 colorectal cancer cell lines with dissimilar initial Cdc42 activity.

**Figure 4:**
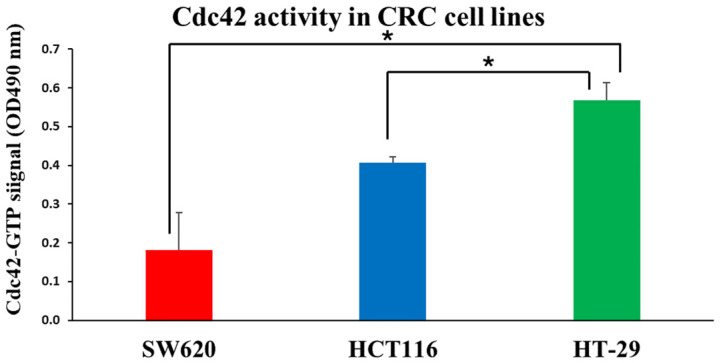
Quantifying active GTP-bound Cdc42 using a Cdc42 G-LISA assay kit on CRC cell lines. Results are expressed as mean ± standard deviation (*n* = 3). (*—*p* < 0.05).

**Figure 5:**
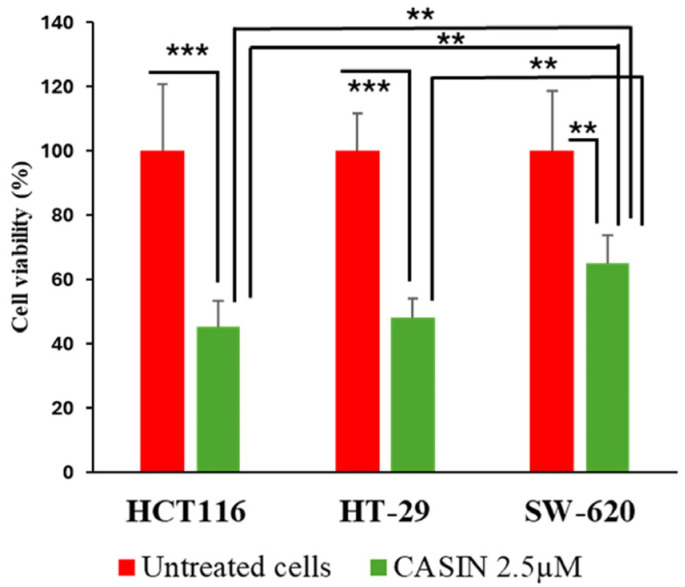
Cell viability was measured using a CCK-8 assay. HCT116, HT-29, and SW620 cells were treated with 2.5 µM of CASIN for 24 h. Statistically significant differences are displayed as ***—*p*  <  0.001, **—*p*  <  0.01. Results are expressed as mean ± standard deviation (*n* = 3).

First, we measured baseline Cdc42 activity in these three cell lines using the G-LISA method. We found that the human colorectal adenocarcinoma cell line HT-29 had the highest activity of Cdc42, followed by the adenocarcinoma cell line HCT116 and SW620 cells (Figure 4). The CCK-8 assay was further utilized to assess the cytotoxicity of unbound CASIN. We found that the SW620 cell line, which exhibited the lowest Cdc42 activity, demonstrated increased resistance to pure CASIN compared to HT-29 and HCT116 cells. (Figure 5). However, despite the varying levels of Cdc42 activity in the HT-29 and HCT116 cancer cell lines, both cell types exhibited identical sensitivity to the Cdc42 inhibitor. This observation suggests that the cytotoxicity of the Cdc42 inhibitor towards cancer cells may also depend on other factors. For instance, it is known that the HT-29 colorectal adenocarcinoma cell line (characterized by mutant p53) differs from HCT116 (characterized by wild-type p53 and microsatellite instability) in terms of apoptosis sensitivity, the degree of chromosomal instability, and other aspects [27,28,29]. HCT116 is known to be a highly aggressive cell line with little or no differentiation capacity, whereas HT-29 has an intermediate differentiation capacity into enterocyte-like cells and expresses mucin [30,31].

**Figure 6:**
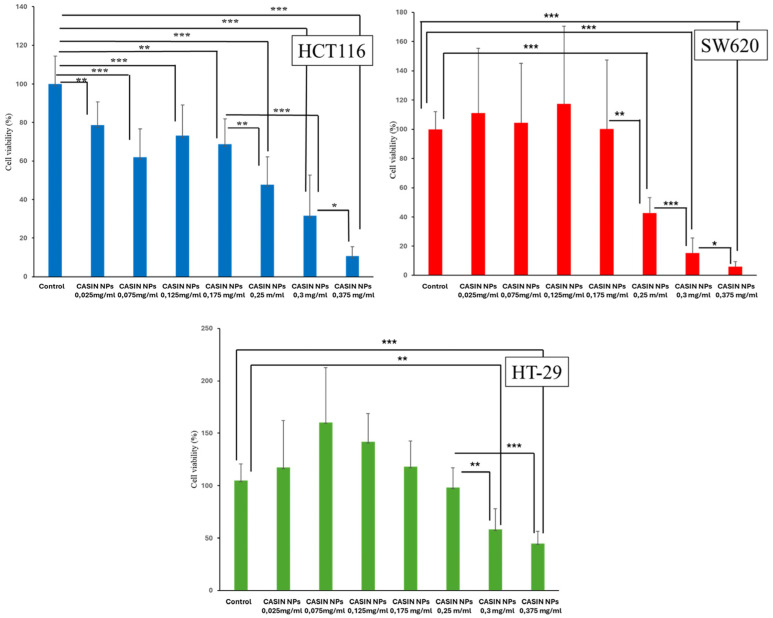
Cell viability was measured using a CCK-8 assay. HCT116, HT-29, and SW620 cells were treated with various concentrations of CASIN-loaded PLGA-PEG-COOH NPs (CASIN NPs) for 24 h. ANOVA results showed significant differences in cell viability between treatment groups: *p* < 0.001 for the HCT116 cell line, *p* < 0.001 for the HT29 cell line, and *p* < 0.001 for the SW620 cell line. Data are presented as means ± standard deviation values (*n* = 3); ***—*p*  <  0.001; **—*p*  <  0.01; *—*p*  <  0.05.

Figure 6 shows the viability of HCT116, HT-29, and SW620 cells after exposure to CASIN-loaded PLGA-PEG-COOH nanoparticles at concentrations varying from 0.025 to 0.375 mg/mL for 24 h. The HCT116 cell line demonstrated the highest nanoparticle sensitivity, exhibiting a dose-dependent decrease in cell viability at all concentrations. Exposure to nanoparticles at concentrations ranging from 0.025 to 0.175 mg/mL reduced cell viability to 68–78%. At nanoparticle concentrations of 0.25–0.3 mg/mL, cell viability decreased to 31–47%. Finally, exposure to the highest concentration of 0.375 mg/mL significantly reduced cell viability to about 10%.

The SW620 cell line responded only to CASIN-loaded PLGA-PEG-COOH nanoparticles at concentrations starting at 0.25 mg/mL. Cell viability decreased from approximately 42% at a concentration of 0.25 mg/mL to around 6% at a concentration of 0.375 mg/mL. The HT-29 cell line showed the lowest responsiveness to CASIN-loaded PLGA-PEG-COOH nanoparticles. The cell viability dropped to 48–57% when exposed to PLGA-PEG-COOH nanoparticles loaded with CASIN at doses of 0.3–0.375 mg/mL, respectively. PLGA-PEG-COOH nanoparticles did not exhibit cytotoxic effects on cancer cells.

The IC50 values for the SW620 and HCT116 lines (9.33 µM and 10.45 µM, respectively) were almost half of the value observed for the HT-29 line (19.55 µM). In analogous nanoparticle systems, IC50 values for various anticancer drugs such as oxaliplatin or fluorouracil encapsulated in PEG-PLGA nanoparticles generally range in the micromolar range depending on the drug, cell line, and experimental conditions, which is in agreement with our findings [32].

Thus, our results suggest that CASIN-loaded PLGA-PEG-COOH nanoparticles exhibit toxicity to all three colorectal cancer cell lines studied, albeit in varying degrees.

In our study, the most sensitive to Cdc42 inhibitor-loaded nanoparticles were the metastatic SW620 and HCT116 cancer cells. As mentioned above, Cdc42 is involved in the progression of colorectal cancer, and its dysregulation at later stages indicates the aggressiveness of the disease [33]. This may be because an impaired regulation of Cdc42 can affect key cell cycle checkpoints, leading to uncontrolled proliferation and promoting the development of CRC tumors [34]. Therefore, inhibiting the proliferation of aggressive CRC tumors by blocking Cdc42 activity to slow tumor growth is one potential therapeutic approach for treating colorectal cancer [12]. In future research, PLGA-PEG-COOH nanoparticles containing CASIN, through improved drug delivery, reduced side effects, and a targeted approach to treatment-resistant and hard-to-treat cancer types, could contribute to developing targeted therapies and advancing personalized medicine.

### 2.3. Hemocompatibility of NPs

Since intravenous injection remains the most widely used method for systemic delivery in clinical and research settings, ensuring rapid and efficient drug distribution [32], we evaluated the compatibility of our nanoparticles with blood. PLGA-PEG nanoparticles are typically biocompatible with blood; however, the impact on blood cells and hemolysis may differ depending on the exact formulation [35,36,37]. For instance, the molecular weight of PEG can significantly influence the hemolytic activity of the polymers [38].

**Figure 7:**
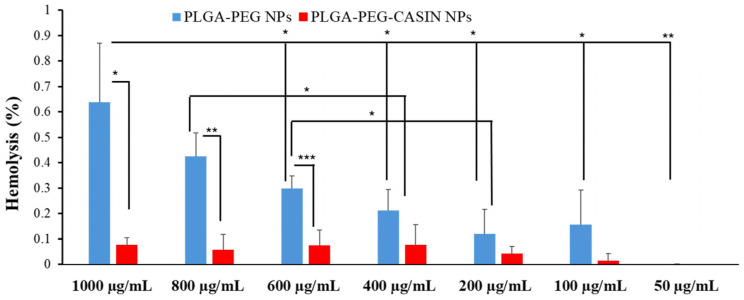
In vitro hemolysis assay of CASIN-loaded PLGA-PEG-COOH NPs and PLGA-PEG-COOH NPs. The presence of hemoglobin in the supernatant was observed at 570 nm. Data are expressed as mean ± SD (*n* = 3); ***—*p*  <  0.001; **—*p*  <  0.01; *—*p*  <  0.05.

Figure 7 demonstrates that empty nanoparticles have a more intense hemolytic impact than CASIN-loaded PLGA-PEG-COOH nanoparticles. Nevertheless, even the highest concentrations of empty nanoparticles do not induce significant hemolysis. CASIN-loaded PLGA-PEG-COOH NPs possess even better blood compatibility, as they have a hemolytic ratio of less than 1% for all tested concentrations, as per ISO/TR 7406 guidelines.

These results indicate that the nanoparticles are safe for systemic administration. Notably, the empty nanoparticles exhibited slightly higher hemolytic activity than the CASIN-loaded nanoparticles, likely due to the improved PLGA-PEG composition, which reduced contact with blood cells while maintaining structural integrity.

## 3. Materials and Methods

### 3.1. Materials

A di-block copolymer of poly(lactide-*co*-glycolide)-block-poly(ethylene glycol)-carboxylic acid endcap (PLGA-PEG-COOH; Mw 20,000:5000 Da; PDI 1.48) was purchased from PolySciTech^®^ Akina Inc. (West Lafayette, IN, USA; catalog number: AI078). 2-[(2,3,4,9-Tetrahydro-6-phenyl-1H-carbazol-1-yl)amino]ethanol (CASIN), dimethyl sulfoxide (DMSO), Poloxamer 407, phosphate buffered saline tablets (PBS), Tween^®^ 80, 1% penicillin/streptomycin, the Cell Counting Kit-8, and Triton-X-100 were purchased from Sigma-Aldrich (Steinheim, Germany). Fetal bovine serum (FBS) and Dulbecco’s modified eagle medium (DMEM) were purchased from Gibco (Waltham, MA, USA). The Cdc42 G-LISA Activation Assay (Colorimetric Format) was purchased from Cytoskeleton, Inc. (Denver, CO, USA). All reagents were of analytical grade and used as supplied. Ultrapure (milli-Q) water was used throughout the experiments involving aqueous solutions.

### 3.2. Preparation of PLGA-PEG-COOH Nanoparticles

Empty and CASIN-loaded PLGA-PEG-COOH nanoparticles were prepared via a one-step nanoprecipitation (single-emulsion) method followed by dialysis with some modification [22]. Briefly, 50 mg of polymer and 4 mg CASIN were dissolved in 1.25 mL of DMSO to form an organic phase and stirred for 1 h. Subsequently, this organic phase was gradually added to 40 mL of 0.5% (*w*/*v*) aqueous Poloxamer 407 solution (used to stabilize nanoparticle suspension) under constant stirring to form a colloidal suspension. The resulting suspension was gently stirred for another 1 h. The suspension was then purified by dialysis against ultrapure water (5 L; changed three times) using a dialysis membrane tube with molecular weight cut-off 12–14 kDa (Sigma-Aldrich, Germany) to eliminate residual solvent. The final product was covered with aluminum foil and stored in a refrigerator for further use.

### 3.3. Dynamic Light Scattering (DLS)

The size, polydispersity index (PDI), and zeta-potential values of both empty and CASIN-loaded PLGA-PEG-COOH nanoparticles were determined using dynamic light scattering (DLS) at a scattering angle of 173° with a Zetasizer Nano-ZS (Malvern Instruments, Malvern, UK). Each nanoparticle suspension was diluted 100-fold with ultrapure water (milli-Q water was additionally filtered through 0.2 μm syringe filters). Measurements employed a refractive index of 1.59 and an absorbance of 0.01, performed in triplicate at 25 °C. The results were reported as average mean ± standard deviation.

### 3.4. Transmission Electron Microscopy (TEM)

Nanoparticles were visualized using a JEOL JEM-1400Plus TEM (Nagoya, Japan) operated at an acceleration voltage of 120 kV. Specimens were prepared by pipetting a 20-fold diluted nanoparticle suspension drop onto parafilm. A glow-discharged holey carbon film-coated 400-mesh copper grid was placed onto the drop with the “carbon” side for 60 sec. The grid was washed by touching its surface with the sample side down on a drop of ultrapure water on parafilm for 60 sec and then blotted dry with filter paper. Next, a drop of 2% (*w*/*v*) uranyl acetate alternative (UAA) solution was applied onto parafilm, and the grid remained in contact with the “carbon” side with UAA for another 60 s. The excess stain was removed by dabbing similarly to the one above, followed by drying it in air prior to TEM characterization. This sample preparation technique was previously reported to give good-quality images for PLGA-PEG nanoparticles [22,23].

### 3.5. Encapsulation Efficiency and Loading Capacity

The Amicon^®^ Ultra-15 centrifugal filter unit with a molecular weight cut-off of 10 kDa was used in these experiments. Each centrifugal filter device was pre-rinsed with 5 mL of PBS (pH 7.40) at 7500 rpm (4088× *g*) and 20 °C for 60 min before further use. The suspension of PLGA-PEG-COOH nanoparticles (5 mL) was then placed in an ultrafiltration tube and centrifuged at 20 °C at 7500 rpm (4088× *g*) for 60 min. The filtrate was discarded, and the retentate was washed with 2 mL of PBS (pH 7.40), followed by further centrifugation at 20 °C at 7500 rpm (4088× *g*) for 60 min. After removing the filtrate, CASIN-loaded nanoparticles in the retentate were disrupted with 2 mL DMSO (left for 6 h at room temperature to dissolve the nanoparticles and CASIN) and further spun at 20 °C at 7500 rpm (4088× *g*) for 60 min. The amount of free CASIN in the supernatant was quantified using a Shimadzu UV-2600i UV/Vis spectrophotometer (Kyoto, Japan), with the absorbance measured at 257 nm (Figure S1). Aliquots were diluted with 0.2% (*v*/*v*) Tween^®^ 80 solution prepared in PBS (pH 7.40). The encapsulation efficiency (EE%) and loading capacity (LC%) were calculated using the following equations:

(1) $$EE\%=\frac{C}{C_{i}}\times 100$$

(2) $$LC\%=\frac{C}{mass\;of\;NPs}\times 100$$

where *C* is the amount of CASIN encapsulated in *NPs*, and *C_i_* is the initial amount of CASIN. A standard curve used to calculate *EE*% and *LC*% can be found in Supplementary Information (Figure S2). All measurements were performed in triplicate. The ultracentrifugation process was performed using an Eppendorf Centrifuge 5810R benchtop centrifuge (Hamburg, Germany).

### 3.6. In Vitro Cumulative Release of CASIN from Nanoparticles

The in vitro cumulative release of CASIN from PLGA-PEG-COOH nanoparticles was studied using a dialysis method adapted from a previously reported protocol [22]. Briefly, 2 mL of CASIN-loaded NPs from stock colloidal solution was transferred in a Pur-A-Lyzer^™^ Maxi 3500 dialysis membrane and immersed in 30 mL of 0.2% (*v*/*v*) Tween^®^ 80 prepared in PBS (pH 7.40) that was then shaken at 80 spm for 72 h at 37 °C. At predetermined intervals, aliquots (5 mL) were withdrawn from the dialysate and replaced with fresh medium to maintain a constant volume. The amount of released CASIN was determined using a Shimadzu UV-2600i UV/Vis spectrophotometer (Kyoto, Japan) (λ_absorbance_ = 257 nm, Figure S1). Figure S2 in Supplementary Information displays the standard curve used in these experiments. All release experiments were conducted in triplicate, and the release kinetics of CASIN from the NPs were also calculated using the zero order model, the first-order model, the Higuchi model, and the Korsmeyer Peppas model.

### 3.7. Cell Culture

Human colorectal cancer (CRC) cell lines HCT116, SW620, and HT-29 were purchased from the American Type Culture Collection (Manassas, VA, USA). Cells were maintained with 10% fetal bovine serum (FBS) and 1% penicillin/streptomycin in Dulbecco’s modified eagle medium (DMEM). Cells were incubated at 37 °C in 5% CO_2_ °C in normoxic conditions.

### 3.8. CCK8-Based Cell Viability Assay

The Cell Counting Kit-8 (CCK-8) assay was used to assess the effect of NPs on the viability of different colorectal cancer cell lines. Briefly, cells were seeded in 96-well plates at a concentration of 5000 cells per well. After 12 h, NPs diluted in culture media (0.025, 0.075, 0.125, 0.175, 0.25, 0.3, and 0.375 mg/mL) were added to the wells and incubated for 24 h. HCT116, SW620, and HT-29 cell lines were also incubated with 2.5 μM CASIN for 16 h. After treatment, 10 μL of the CCK-8 reagent was added to each well and further incubated for 1 h, followed by optical density measurement at 450 nm using a Synergy Hybrid H1 Microplate Reader (Biotek, Shoreline, WA, USA). The IC50 value was calculated via a four-parameter logistic regression model. The computations were conducted using an online tool developed by AAT Bioquest, Inc., based in Sunnyvale, CA, USA. The tool may be accessed at https://www.aatbio.com/tools/ic50-calculator (accessed on 10 September 2024).

### 3.9. G-Lisa Assay

The Cdc42 G-LISA activation assay (colorimetric format) (was used to determine the active GTP-bound form of Cdc42, according to the manufacturer’s instructions. The basal activities of Cdc42 in HCT116, SW620, and HT-29 cell lines treated by CASIN or PLGA-PEG-COOH NPs with and without CASIN were determined at λ_absorbance_ = 490 nm using a Synergy Hybrid H1 Microplate Reader (Biotek, USA).

### 3.10. Hemolysis Assay

The in vitro hemolysis assay was performed on human erythrocytes. All procedures related to the blood collection were performed according to the protocols approved by the Local Ethics Committee of National Laboratory Astana (Registration number IORG 0006963, N02-2022, 01.04.2022). Briefly, blood samples were collected from healthy volunteers in K2-EDTA vacutainers and centrifuged at 500× *g* for 10 min. After several PBS washes, the plasma from the fresh blood sample was removed, and red blood cells (RBCs) were diluted to prepare a 4% (*v*/*v*) RBC solution in PBS. Subsequently, 800 μL of RBC solution in separate Eppendorf microtubes was mixed with either 200 μL of PBS (used as a negative control), 200 μL of NP solution (50, 100, 200, 400, 600, 800, and 1000 µg/mL) prepared in PBS, or 200 μL of 1% Triton-X-100 (used as a positive control). The mixture was then incubated for 2 h at 37 °C with mild stirring breaks every half hour. Following incubation, the mixture was centrifuged for 10 min at 2000× *g*. Then, 100 μL of the supernatant was taken into the 96-well plates. The absorbance (OD) at 570 nm was determined using a Synergy Hybrid H1 Microplate Reader (Biotek, USA). The percentage of hemolysis was calculated using the following equation:

(3) $$\%\;hemolysis=\frac{{Absorbance}_{sample}-{Absorbance}_{negative\;control}}{{Absorbance}_{positive\;control}-{Absorbance}_{negative\;control}}\times 100$$

### 3.11. Statistical Analysis

Data are expressed as means ± standard deviations (SDs) of a minimum of three independent experiments. Data were analyzed using Student’s *t*-test for comparison between two independent groups and ANOVA for comparison among three or more groups in Microsoft Excel Microsoft 365(Microsoft Corp., Redmond, WA, USA) software. *p* values of ≤0.05 were considered to indicate statistical significance. A one-way ANOVA was conducted to compare the effect of different concentrations of CASIN-loaded PLGA-PEG-COOH nanoparticles on cell viability in three different cell lines (HCT116, HT29, and Sw620). A post-hoc Tukey’s Honest Significant Difference (HSD) test was performed to further explore the differences between specific groups.

## 4. Conclusions

In the present work, we report the design of a novel Cdc42 inhibitor nanoformulation by encapsulating the Cdc42 inhibitor CASIN within PLGA-PEG-COOH nanoparticles. Our study demonstrates that CASIN NPs dose-dependently inhibit cancer cell proliferation in vitro. The IC50 values for CASIN-loaded nanoparticles are comparable to those of other nanoparticle-based cancer therapies, further highlighting their efficacy as a promising treatment option. In addition, the CASIN-loaded PLGA-PEG-COOH NPs showed a favorable hemocompatibility profile, indicating their potential safety for systemic administration. The findings validate the efficacy of CASIN-loaded PLGA-PEG-COOH NPs as a promising targeted treatment for colorectal cancer. Our results further emphasize the need for more investigation into the function of Cdc42 in the progression of colorectal cancer and the mechanisms governing the control of Cdc42 and oncogenic signaling pathways.

Further research will focus on animal studies, including the pharmacokinetics, toxicity, and antitumor activity of CASIN-loaded PLGA-PEG nanoparticles. Such studies will advance our knowledge of medication distribution, retention, and safety characteristics. To tackle the difficulties posed by tumor heterogeneity and resistance, forthcoming research will prioritize optimizing the composition of nanoparticles loaded with CASIN.

Additionally, efforts could be made to formulate these nanoparticles in conjunction with other therapeutic agents to augment the anti-cancer effects through synergistic mechanisms. One of the promising approaches here is to explore the combination of Cdc42 inhibitors with chemotherapeutic agents commonly used for CRC treatment, such as fluorouracil and oxaliplatin. This combination could enhance the anti-cancer effects through synergistic mechanisms. Overall, our multidisciplinary approach, which integrates knowledge from materials science, oncology, and nanomedicine disciplines, signifies progress in advancing novel nanotechnologies for managing colorectal cancer and paves the way for further investigations in this field.

## Figures and Tables

**Table 1:** Physicochemical characteristics of PLGA-PEG-COOH nanoparticles.

Formulation	Mean Diameter (nm)	PDI	Zeta-Potential (mV)	EE%	LC%
PLGA-PEG-COOH	171 ± 2	0.076	–41 ± 1	N/A	N/A
PLGA-PEG-COOH with CASIN	86 ± 1	0.104	–30 ± 1	66 ± 5	5 ± 1

PLGA-PEG-COOH, poly(lactide-*co*-glycolide)-*block*-poly(ethylene glycol)-carboxylic acid endcap; PDI, polydispersity index; EE%, encapsulation efficiency; LC%, loading capacity; N/A, not applicable. Results are expressed as mean ± standard deviation (*n* = 3).

## Data Availability

The original contributions presented in the study are included in the article/Supplementary Materials, further inquiries can be directed to the corresponding author.

## Supplementary Materials

The following supporting information can be downloaded at: https://www.mdpi.com/article/10.3390/pharmaceutics16101301/s1, Figure S1: UV/Vis spectra of CASIN at various concentrations recorded in 0.2% (v/v) Tween^®^ 80 prepared in PBS (pH 7.40). The absorbance wavelength used is 257 nm; Table S1.Physicochemical characteristics of PLGA-PEG-COOH nanoparticles prepared in different batches; Figure S2: TEM microphotographs of PLGA-PEG-COOH nanoparticles: empty (A, B) and CASIN-loaded (C, D); Figure S3: A standard curve used to determine the amount of CASIN in EE%, LC% and in vitro cumulative release; Figure S4: Fitting of the release kinetic of CASIN from the nanoparticles with different models.
