# Supplementary material for: PLGA-PEG Nanoparticles Loaded with Cdc42 Inhibitor for Colorectal Cancer Targeted Therapy
Source: Pharmaceutics. 2024 Oct 6;16(10):1301. doi: 10.3390/pharmaceutics16101301 (PMC11510643; doi:10.3390/pharmaceutics16101301)
Supplement: Supplementary file 1 [file pharmaceutics-16-01301-s001.zip › pharmaceutics-3169979-supplementary.pdf]

Supplementary Materials: PLGA-PEG nanoparticles loaded with Cdc42 inhibitor for colorectal cancer targeted therapy

Sanazar Kadyr [2], Altyn Zhuraliyeva [1], Aislu Yermekova [1], Aigerim Makhambetova [1], Daulet B. Kaldybekov [3], Ellina A. Mun [4], Denis Bulanin [2], Sholpan N. Askarova [1] and Bauyrzhan A. Umbayev [1], *

1 Laboratory of Bioengineering and Regenerative Medicine, National Laboratory Astana, Nazarbayev University, 010000 Astana, Kazakhstan
2 School of Medicine, Nazarbayev University, 010000 Astana, Kazakhstan
3 Department of Chemistry and Chemical Technology, Al-Farabi Kazakh National University, 050040 Almaty, Kazakhstan
4 School of Sciences and Humanities, Nazarbayev University, 010000 Astana, Kazakhstan

Table S1. Physicochemical characteristics of PLGA-PEG-COOH nanoparticles prepared in different batches.

Batch number	Formulation	Mean diameter (nm)	PDI	Zeta-potential (mV)	EE%	LC%
I	PLGA-PEG-COOH	156 ± 2	0.052	–32 ± 1	N/A	N/A
	PLGA-PEG-COOH with CASIN	86 ± 1	0.097	–7.0 ± 1	69.9	5.60
II	PLGA-PEG-COOH	176 ± 2	0.085	–30 ± 1	N/A	N/A
	PLGA-PEG-COOH with CASIN	83 ± 1	0.130	–28 ± 3	59.87	4.79
III	PLGA-PEG-COOH	181 ± 1	0.069	–44 ± 1	N/A	N/A
	PLGA-PEG-COOH with CASIN	87 ± 1	0.141	–29 ± 1	67.50	5.40

PLGA-PEG-COOH, poly(lactide-*co*-glycolide)-*block*-poly(ethylene glycol)-carboxylic acid endcap; PDI, polydispersity index; EE%, encapsulation efficiency; LC%, loading capacity; N/A, not applicable.

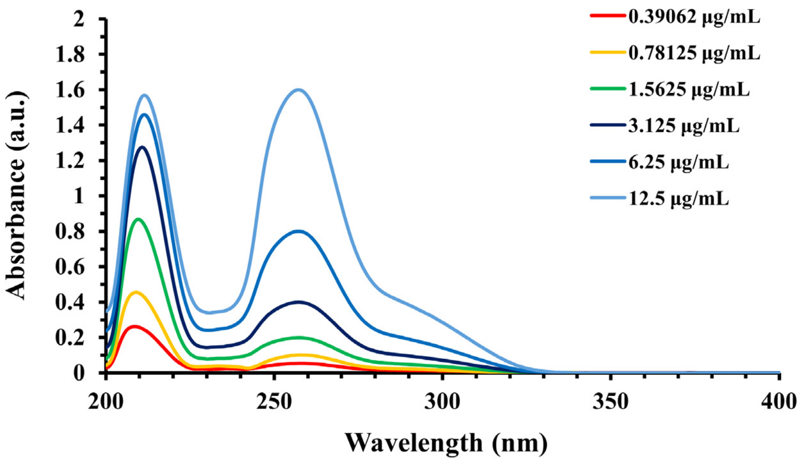

Figure S1. UV/Vis spectra of CASIN at various concentrations recorded in 0.2% (v/v) Tween® 80 prepared in PBS (pH 7.40). The absorbance wavelength used is 257 nm.

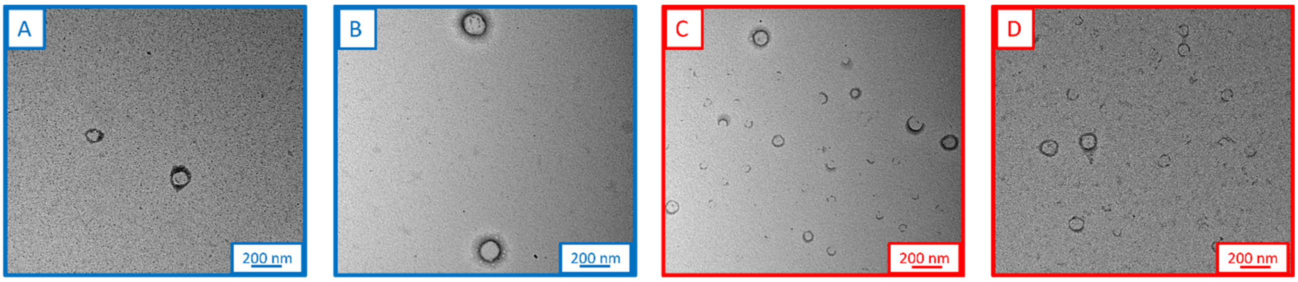

Figure S2. TEM microphotographs of PLGA-PEG-COOH nanoparticles: empty (A, B) and CASIN-loaded (C, D).

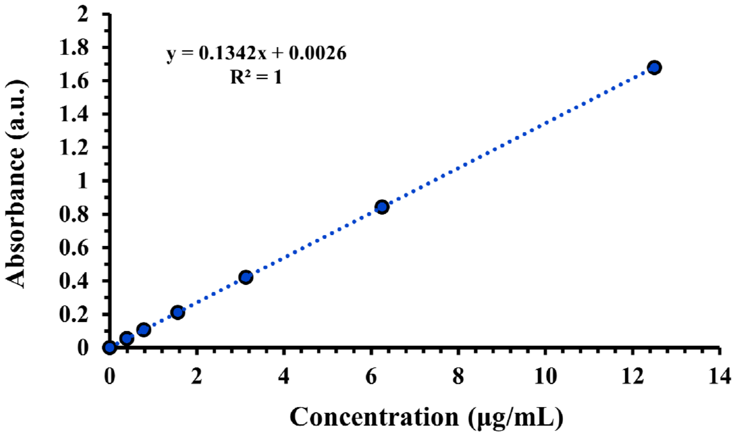

Figure S3. A standard curve used to determine the amount of CASIN in EE%, LC% and *in vitro* cumulative release.

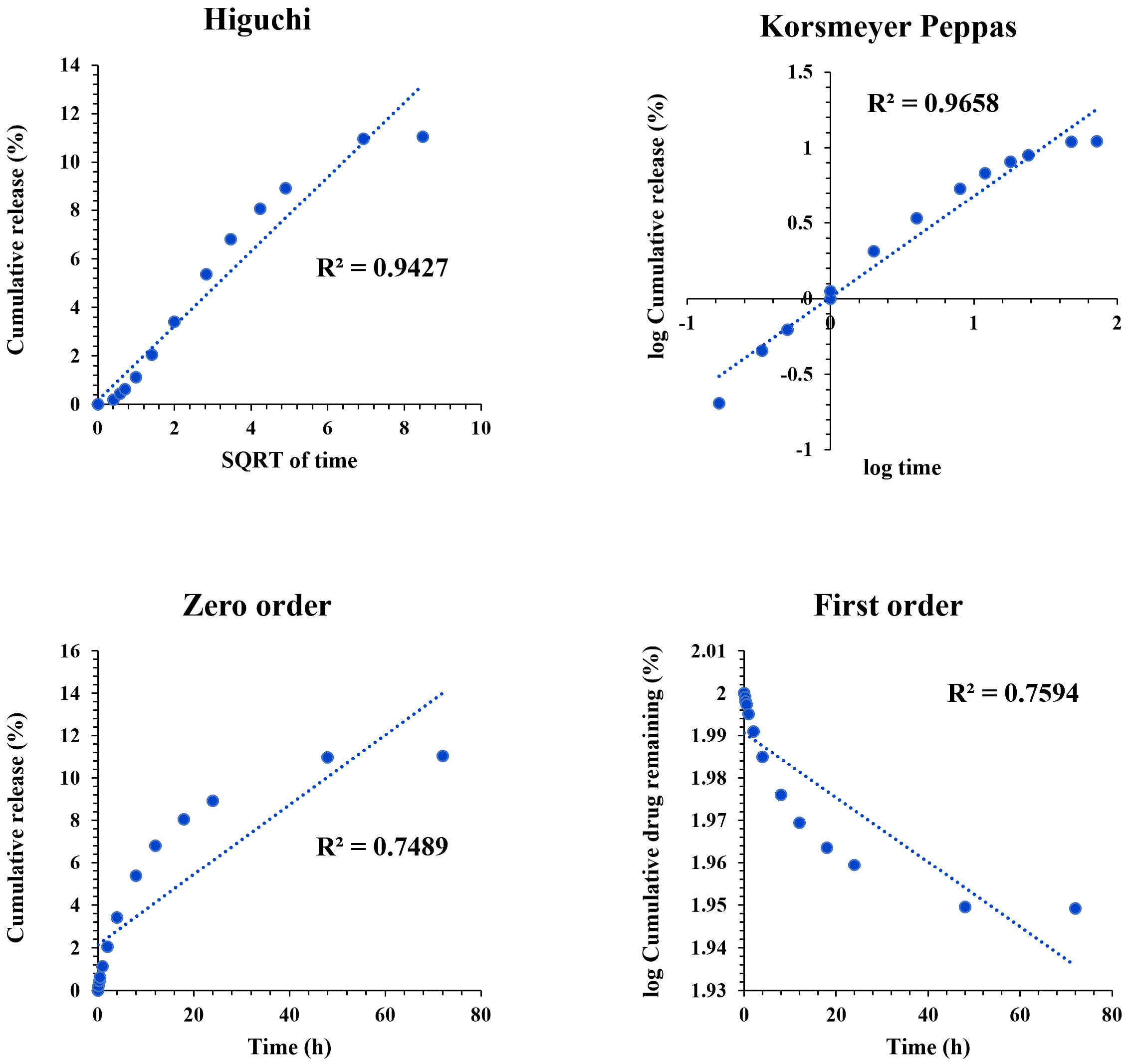

Figure S4. Fitting of the release kinetic of CASIN from the nanoparticles with different models.
